# Comment on “Clinical and Pathological Characteristics of Autoimmune Hepatitis with Acute Presentation”

**DOI:** 10.1155/2020/3895375

**Published:** 2020-10-23

**Authors:** Paolo Muratori

**Affiliations:** Department of Science for the Quality of Life (QUVI), University of Bologna, Bologna, Italy

I read with interest the recent paper by Shen et al. on the acute presentation of autoimmune hepatitis (AIH) in a Chinese population [1]. In the same period, my colleagues and I published a similar study on a well-characterized cohort of Italian patients [2]; in particular, both we and Shen et al. used identical selection criteria to define the acute onset of the disease (ALT and AST >X10 upper normal limit and/or total bilirubin >5 mg/dl), and this makes it possible to compare the two experiences.

Similar to Shen et al., we observed a higher histological grading in acute patients with respect to the chronic counterpart, but in contrast, the Chinese researchers found significantly higher IgG serum levels in acute patients than in chronic ones. With respect to IgG, we did not find any difference between the two populations studied. This difference is difficult to explain since hyper-IgG is often absent in acute onset of the disease. A possible explanation could reside in some “acute on chronic” cases enrolled in the acute group, while in our experience, we analysed the “genuine” acute onset with respect to the “acute on chronic” type. Under the serological profile, Shen et al. reported a significantly higher titre for ANA in the acute group, while we did not find any difference in terms of titre or frequency of autoantibodies in our experience. Curiously, Shen et al. did not report the frequency of smooth muscle antibodies in their patients.

The most intriguing result from our study was the significantly better prognosis of acute patients with respect to the chronic ones, but this result cannot be compared with the Chinese authors' experience since their study lacks a follow-up. We agree, however, with Shen et al. on the need to perform a multicentre study not only to detect further diagnostic tools for the disease but also to compare the different courses of the disease with respect to the geographical area.
